# Potentially malignant oral disorders in patients with fanconi anemia: A cross-sectional study

**DOI:** 10.1016/j.htct.2026.106462

**Published:** 2026-05-07

**Authors:** Joana Leticia Vendruscolo, Bárbara Soldatelli Ballardin, Juliana Lucena Schussel, Cassius Carvalho Torres-Pereira

**Affiliations:** Department of Stomatology, Federal University of Parana. *Av*. Lothário Meissner, 632 Jardim Botânico, Curitiba, Paraná CEP: 80210-170, Brazil

**Keywords:** Fanconi anemia, Oral leukoplakia, Oral cancer, Hematopoietic stem cell transplantation

## Abstract

**Introduction:**

Patients diagnosed with Fanconi Anemia present multiple congenital anomalies, pancytopenia, and defective DNA repair mechanisms, making them more susceptible to malignant neoplasia development, with notably higher incidence of oral squamous cell carcinoma. Hematopoietic stem cell transplantation is the definitive treatment for the hematological manifestations but it is considered a factor that significantly increases the risk of malignant transformation. Oral potentially malignant disorders frequently precede the development of oral carcinoma. Given the specific characteristics of this population, there is a critical need to strengthen surveillance programs and screening strategies for these lesions. The aim of this study was to evaluate the overall prevalence of different oral potentially malignant disorders in patients with Fanconi Anemia, without distinguishing among specific subtypes.

**Methods:**

This was an observational cross-sectional study conducted with individuals who attended the Dental Clinic in the Onco-Hematology department of the Hospital de Clínicas Complex, Federal University of Paraná from March 2022 to January 2024. Demographic and clinical data were collected from patients with no history of oral squamous cell carcinoma.

**Results:**

The study involved 110 patients of both genders (51.91% men and 49.09% women), with a mean age of 19.96 years. Of these, 40.9% presented at least one oral potentially malignant disorder; the presence of these disorders was significantly associated with having undergone hematopoietic stem cell transplantation. Older patients also had a higher risk of developing these lesions.

**Conclusions:**

These disorders should be regarded as distinct entities in this population, with the risk of malignant transformation potentially being higher than in the general population not affected by Fanconi Anemia.

## Introduction

Fanconi Anemia (FA) is a rare autosomal recessive disorder that affects approximately 1 in 360,000 individuals [1,2]. Typically diagnosed in early childhood due to the presence of multiple congenital anomalies and severe pancytopenia, which can progressively result in bone marrow failure [3,4]. Moreover, these individuals exhibit defective DNA repair mechanisms, a critical system responsible for maintaining genome integrity and preventing malignancies [5]. This defect is considered the primary reason for the increased susceptibility to cancer development in individuals with FA [6], particularly acute myeloid leukemia and solid tumors, including oral squamous cell carcinoma (OSCC) [3], which currently stands as the leading cause of mortality in this population.

Hematopoietic stem cell transplantation (HSCT), the definitive treatment for the hematological manifestations of FA, is considered a factor that significantly increases the risk of malignant transformation [7, 8, 9], with an earlier onset compared to individuals who have not undergone HSCT [10].

Oral potentially malignant disorders (OPMDs) may precede the development of OSCC. Oral leukoplakia (OL), defined as a “predominantly white patch/plaque that cannot be rubbed off” [11], is among the most common OPMD observed in the oral cavity of FA patients [7]. These lesions, as well as red lesions (oral erythroplakia [OE]) or mixed red-and-white lesions (oral erythroleukoplakia [OLE]), are associated with a risk of malignant transformation [11, 12, 13, 14, 15], underscoring the importance of regular follow-up visits and systematic oral screening in FA patients [13].

Among individuals who have undergone HSCT, those who develop chronic graft-versus-host disease (chronic GvHD), a complication arising from alloimmune and autoimmune mechanisms following transplantation, face an even higher risk of malignant transformation [9,16,17]. Clinically, chronic oral GvHD presents with lichenoid features, which may or may not be accompanied by areas of erythema and ulceration and is considered a distinct OPMD entity, separate from OL [11,18]. Histopathologically, the diagnosis of oral chronic GvHD is based on the presence of a lichenoid interface inflammatory infiltrate with lymphocytic exocytosis and variable epithelial apoptosis [18,19], findings that differ from those observed in OL [20,21].

The aim of this study was to identify potential factors associated with the presence of OPMDs in general, without differentiating among OL, OE, OLE, or chronic GvHD, in FA individuals. By identifying patients at increased risk for malignant transformation, this study seeks to support the development of more effective and targeted monitoring and treatment strategies for this population.

## Materials and methods

### Study design

This was a cross-sectional observational study that was approved by the ethics committee of the Hospital de Clínicas of the Federal University of Paraná (CAAE: 53,235,921.3.0000.0096), and conducted in the dental office of the Onco-Hematology department of the same Hospital between March 2022 and January 2024.

The inclusion criteria for this study comprised patients with a confirmed diagnosis of FA who had attended at least one consultation during the study period. Written informed consent or assent was obtained from all participants prior to their inclusion. Patients were excluded if they had a history of previously diagnosed OSCC or if OSCC was present at the time of the evaluation.

### Data collection

Demographic and historic data were obtained through anamnesis and electronic hospital records. Relevant information included age, sex, ethnicity, state of residence, and harmful health habits (use of substances such as alcohol, conventional, or electronic cigarettes), as well as information about HSCT (whether it was performed, the year and type of HSCT). Information regarding donor sex and chemotherapy conditioning for HSCT was not considered in this study.

A thorough physical examination of the oral mucosa was conducted by two dentists with expertise in stomatology.

**Fig. 1:**
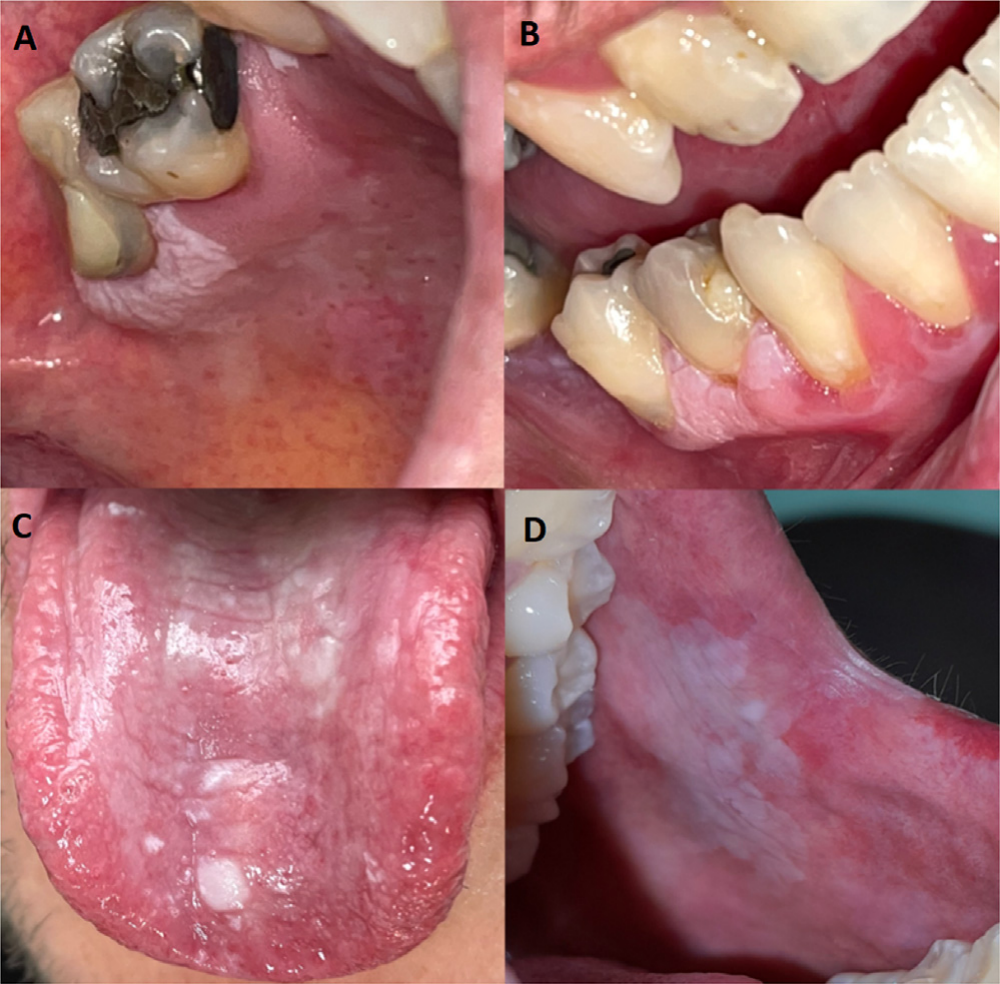
Clinical presentation of oral potentially malignant disorders (OPMDs).

The following conditions were considered OPMDs: OL, OE, OLE and ‘lichen planus-like’ features (Figure 1). All of these conditions were analyzed collectively as a single group, referred to as OPMD.

Representative clinical images illustrating the diversity of OPMDs: (A) Oral leukoplakia (OL) presenting as a well-defined white plaque on the palate; (B) Oral erythroleukoplakia (OLE) affecting the gingival mucosa, showing a mixed red and white appearance; (C) ‘Lichen planus-like’ features on the dorsal and lateral surfaces of the tongue, characterized by reticular and plaque-like white lesions; (D) Oral erythroplakia (OE) on the buccal mucosa, presenting as a predominantly erythematous area with subtle white components

### Statistical analysis

Statistical analyses were performed using the RStudio software (R4.4). Participants were initially stratified into two groups based on the primary outcome: the presence or absence of OPMDs. Descriptive data analysis was employed to define the general characteristics of the sample. Means and standard deviations were calculated for normally distributed variables, while medians and interquartile ranges were used for variables with non-normal distributions. Categorical variables were described using absolute numbers and percentages.

The potential association between HSCT and the presence of OPMDs was evaluated using univariate and multivariate logistic regression. The multivariate model was adjusted for sex, age, and harmful habits (use of substances such as tobacco, electronic cigarettes, and alcohol). Sensitivity analysis was performed to assess the strength of the association and the influence of covariates. Additionally, among individuals who had undergone HSCT, a separate analysis was performed to investigate the relationship between the time since transplantation and the appearance of OPMDs.

## Results

A total of 120 patients diagnosed with FA and no history of OSCC were evaluated. During the initial physical examination, ten patients were found to have lesions compatible with OSCC, all of which were confirmed through histopathological analysis leading to the exclusion of these individuals from the study. Thus, a total of 110 patients, with a mean age of 19.67 years, were included in the statistical analysis. The cohort compromised 56 men (51.91%) and 54 women (49.09%). Among the participants, 45 individuals (40.91%) presented at least one OPMD. When analyzed by age and HSCT status, statistically significant differences (p-value <0.05) were observed between the groups with and without OPMD.

**Fig. 2:**
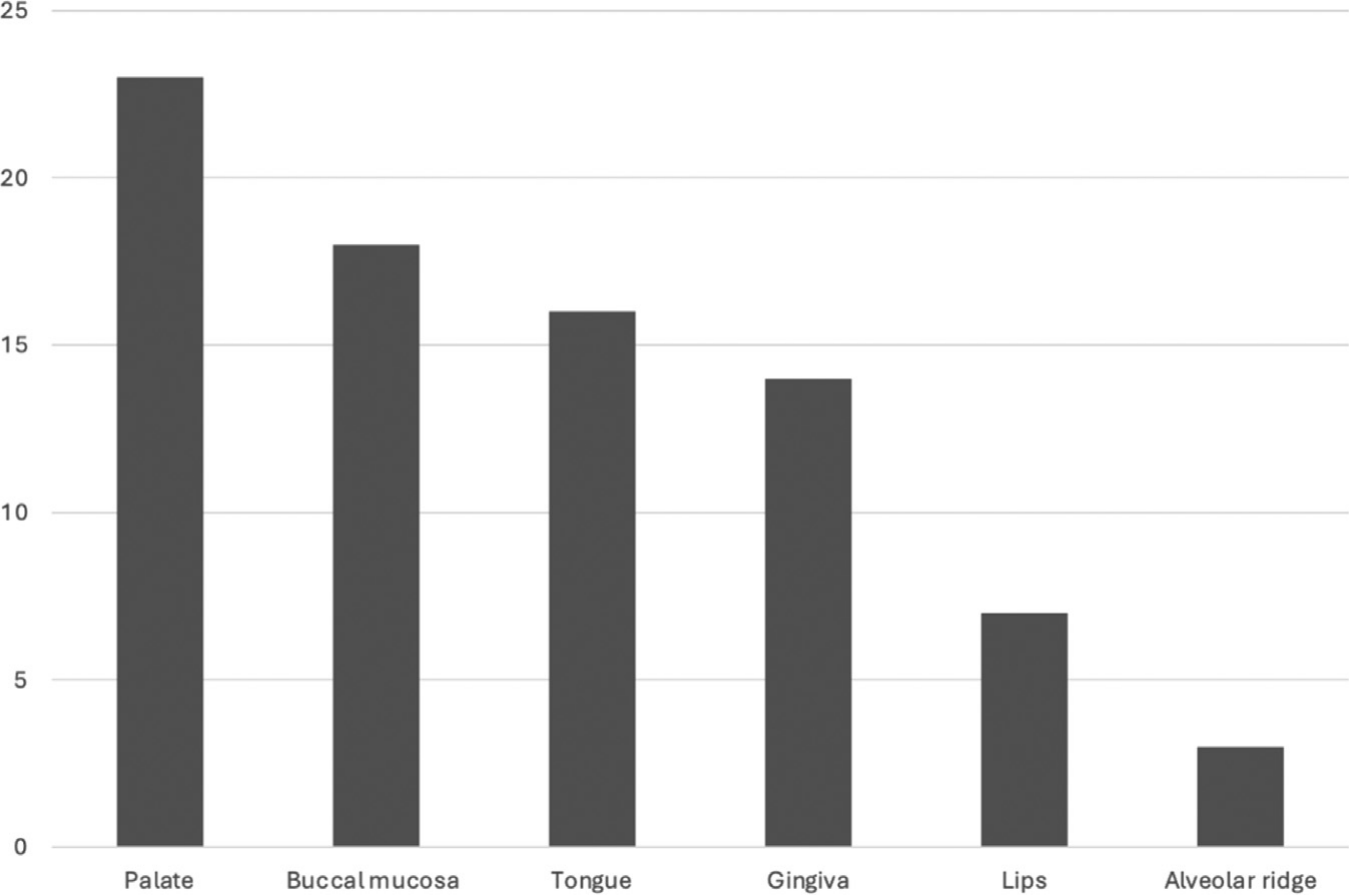
Distribution of oral potentially malignant disorders (OPMDs) by anatomical site. Frequency of OPMD occurrence across different regions of the oral cavity.

The general characteristics of the sample are summarized in Table 1, while the distribution of OPMDs according to anatomical site within the oral cavity is illustrated in Figure 2.

**Table 1:** General characteristics of the sample. The values represent the proportion of cases in each category according to the presence or absence of oral potentially malignant disorders.

	OPMD		p-value
	Absent	Present	
n	65	45	
Men – n (%)	28 (43.1)	28 (62.2)	0.075
Women - n (%)	37 (33.6)	17 (15.4)	
Age (years)*	17.17 (7.49)	23.29 (7.82)	**<0.001**
Geographical region - n (%)			0.785
Midwest	5 (7.7)	6 (13.3)	
Northeast	21 (32.3)	17 (37.8)	
North	4 (6.2)	2 (4.4)	
Southeast	13 (20.0)	8 (17.8)	
South	22 (33.8)	12 (26.7)	
Non-White - n (%)	47 (72.3)	28 (62.2)	0.364
White - n (%)	18 (16.4)	17 (15.4)	
HSCT – n (%)	43 (66.2)	40 (88.9)	0.012
Time after HSCT (years)⁎⁎	7 (2.00–12.00)	9.50 (6.00–18.25)	0.014
Age at HSCT (years)⁎⁎	9.50 (7.75–12.00)	9.50 (7.75–14.00)	0.605
Type of HSCT - n (%)			
Matched related HSCT	19 (43.2)	17 (41.5)	1.000
Unrelated HSCT	16 (36.4)	10 (24.4)	0.336
Umbilical cord HSCT	1 (2.3)	2 (4.9)	0.950
Haploidentical HSCT	9 (20.5)	11 (26.8)	0.662
Harmful health habits - n (%)	1 (1.5)	4 (8.9)	0.176

HSCT: Hematopoietic Stem-Cell Transplantation.

⁎ The mean and standard deviation were used for variables with a normal distribution.

⁎⁎ The median and interquartile range were applied to continuous variables with non-normal distributions.

The association between the presence of OPMDs and HSCT was found to be statistically significant in the univariate regression model, indicating that individuals who had undergone HSCT were up to four times more likely to present OPMDs compared to those who had not undergone the procedure. In the multivariate model, HSCT and age were identified as significant predictive variables for the presence of OPMDs (Table 2).

**Table 2:** Association between hematopoietic stem cell transplantation and oral potentially malignant disorders (Logistic Regression).

	OR		95% CI	p-value
**Univariate model**				
HSCT	4.09	1.41 - 11.84		**0.009**
**Multivariate model**				
HSCT	6.23	1.55 - 25.04		**0.01**
Age	1.12	1.05 - 1.19		**< 0.001**
Sex	2.46	0.98 - 6.17		0.054
Harmful health habits	7.38	0.55 - 98.98		0.131

OR: Odds Ratio; 95% CI: 95% confidence interval; HSCT: Hematopoietic Stem-Cell Transplantation.

Of the individuals who had undergone HSCT, the time since transplantation showed an association with the presence of OPMDs in the univariate model (p-value <0.05). However, after adjustment in the multivariate model, this variable lost statistical significance (p-value >0.05), while the individual's age remained a significant factor. This finding suggests that age is a predictive variable for the presence of OPMDs and may act as a confounding factor, explaining the apparent significance of time since HSCT observed in the univariate model (Table 3).

**Table 3:** Association between oral potentially malignant disorders and time after hematopoietic stem cell transplantation (Logistic Regression).

	OR	95% CI	p-value
**Univariate model**			
Time post-HSCT	1.11	1.03 – 1.2	**0.009**
**Multivariate model**			
Age	1.1	1.02 - 1.19	**0.013**
Sex	1.72	0.82 – 5.8	0.11
Harmful health habits	1.06	0.96 – 1.16	0.23

OR: Odds Ratio; 95% CI: 95% confidence interval; HSCT: Hematopoietic Stem-Cell Transplantation.

After conducting the sensitivity analysis, age and the occurrence of HSCT remained statistically significant predictors across all models (Table 4).

**Table 4:** Sensitivity analysis.

	OR	95% CI	p-value
Model 1			
HSCT	4.12	1.3–13	**0.016**
Model 2			
HSCT	4.12	1.3–13	**0.016**
Age	1.11	1.05–1.18	**<0.001**
Model 3			
HSCT	4.19	1.29–13.61	**0.017**
Age	1.12	1.06–1.2	**<0.001**
Sex	2.85	1.16–7.02	0.022

OR: Odds Ratio; 95% CI: 95% confidence interval; HSCT: Hematopoietic Stem-Cell Transplantation.

## Discussion

The life expectancy of individuals with FA has improved in recent years, largely due to advances in HSCT, which have led to better outcomes in the treatment of bone marrow failure [13]. As a result, another major challenge faced by this population has gained increasing importance: the incidence of malignant neoplasms.

The incidence of OSCC in individuals with FA is up to 700 times higher compared to individuals without the disease matched for age and sex [22]. Moreover, unlike the general population, cancer in FA patients typically manifests in young adults of around 26 years of age [22,23], with a slight predominance in women (2:1) [24,25].

Regarding the tumor site, the lateral border of the tongue is the most commonly affected site, followed by the gingiva, buccal mucosa, and retromolar trigone. Furthermore, FA patients have a higher tendency to develop multiple tumors [25,26]

In general, oral lesions are highly prevalent in the post-transplant period. Many of these lesions are traumatic, frictional, or infectious in nature, and as their potential for malignant transformation is not in question [11], these patients were excluded from this study similar to previous studies [27]. However, some lesions develop independently and may carry a risk of malignant transformation over time. In this study, the classification of OPMDs was based on clinical features after the exclusion of differential diagnoses. Biopsies were recommended only in carefully selected cases, in accordance with standard practice, particularly given that these individuals are already under continuous and intensive medical care. Consequently, the number of patients who underwent histopathological examinations was limited. To be classified as an OPMD, lesions had to be non-removable by scraping and have no identifiable traumatic cause, thereby distinguishing them from frictional keratosis. This approach adhered to the World Health Organization (WHO) criteria for defining leukoplakia and erythroplakia [11,28].

It is also important to emphasize that, in the clinical practice of this population, professionals face significant challenges in distinguishing oral manifestations and accurately identifying lesions during OPMD screening. The clinical examination in FA is often complicated by microstomia [29] with this difficulty being further exacerbated in the post-HSCT setting. It is not uncommon for patients to present with generalized oral mucosal erythema, whitish or brownish striae with a lichen planus-like appearance, and multiple overlapping lesions, making it difficult to determine which lesions should be considered potentially malignant.

These features often differ clinically from the classic OPMDs (OL, OE, OLE) and may closely resemble chronic oral GvHD [19,20,28]. Given the wide spectrum of oral manifestations, the frequent coexistence of overlapping lesions (particularly after HSCT), and the common absence of histopathological confirmation of chronic oral GvHD, this present study focused exclusively on the overall clinical diagnosis of OPMDs, without distinguishing between specific subtypes. Nevertheless, the importance of carefully assessing all oral alterations during the oral examination of this unique population must be emphasized, as a comprehensive and systematic evaluation is crucial for appropriate monitoring and early detection of lesions with malignant potential.

In addition to their heightened susceptibility to malignancies, the prognosis of OSCC in FA patients is particularly concerning. Treatment is typically limited to surgical tumor removal due to the pronounced hypersensitivity these patients exhibit to chemotherapy and radiotherapy, which are generally reserved for more advanced cases with a poorer prognosis [6,26]. Consequently, screening for OPMDs emerges as a key strategy for preventing OSCC in this population. Understanding the potential factors associated with the presence of these oral lesions represents a crucial first step toward enhancing clinical decision-making and optimizing treatment strategies.

A significant percentage of evaluated patients (40.91%) presented with at least one OPMD. The most affected site for OPMDs was the palate (15.97%), corroborating previous findings by Grein-Cavalcanti et al [7]. Interestingly, the buccal mucosa was the second most affected site for OPMDs in this population, aligning with the findings of Mattos et al. who reported a case series of oral cancer at this site [30]. In contrast, studies on the general population without FA indicate that leukoplakia more frequently develops on the lateral/ventral tongue and buccal mucosa, with the palate being an uncommon site [11,12].

The prevalence of OPMDs observed in this study is higher than that reported by other authors who specifically evaluated the prevalence of leukoplakia. For example, Grein-Cavalcanti et al. reported a prevalence of 12%, however, their sample was younger and only included patients who had not undergone HSCT [7]. When considering patients without HSCT in the current study, the prevalence of OPMDs drops to 4.54%, further highlighting the impact of HSCT on the development of lesions. Similarly, Archibald et al. reported a rate of 8.6% for leukoplakia though their study population had a mean age of 11.3 years, which is notably younger than the population in the present study [27]. These differences underscore the importance of age and treatment history in the prevalence of OPMDs among FA patients.

HSCT, along with patient age, was identified as a significant factor associated with the presence of OPMDs. It is already described in the literature that patients undergoing this hematological treatment have a higher prevalence of OSCC [15], and tend to develop the disease at an earlier age compared to those who do not undergo HSCT [10,13,26]. A similar trend seems to occur with OPMDs [31], which may explain the differences in the prevalence of leukoplakia observed in studies evaluating individuals prior to HSCT [7]. One possible explanation for the increased frequency of OPMDs after transplantation is the development of chronic oral GvHD, which may create a microenvironment conducive for epithelial dysplasia and malignant transformation [19,20]. Although chronic GvHD was not analyzed separately in the present study due to the absence of systematic histopathological confirmation, lesions exhibiting lichenoid characteristics were included within the overall OPMD group. Therefore, it is possible that some of the lesions classified as OPMDs in this study may in fact represent manifestations of chronic oral GvHD. This diagnostic overlap reflects the inherent challenges in clinically distinguishing these entities in patients with FA, particularly in the post-HSCT setting.

In the univariate analysis, variables such as sex, ethnicity, and region of origin were not statistically significant and showed no association with OPMD prevalence. However, previous studies suggest that women may have a higher risk of leukoplakia malignancy [32]. Regarding HSCT, the type of transplant did not appear to influence oral lesion development. Future studies focusing on the degree of oral mucositis and chemotherapeutic agents used during HSCT conditioning may provide valuable insights into their long-term effects on oral lesion development.

Tobacco and alcohol consumption were not associated with OPMDs in this study, likely due to the young age of the cohort; OPMDs related to these risk factors typically manifest in older individuals after prolonged exposure. Moreover, it is important to note that in a non-FA population, both alcohol and tobacco consumption do not appear to influence malignant transformation of leukoplakia [11,12].

Some limitations of this study include its cross-sectional design, which precludes the assessment of the progression of OPMDs over time, and the lack of histopathological diagnoses to assess the presence of tissue dysplasia and chronic oral GvHD [20,32]. Longitudinal studies separating OPMDs into their variations with rigorous classification are necessary to understand the risk of malignant transformation of these lesions [33].

## Conclusions

This study provides important data on the overall prevalence of OPMDs in individuals with FA, a population known to be at particularly high risk for malignant transformation. The findings of this study show that OPMDs are more prevalent among young adults who have undergone HSCT, regardless of sex or time since transplantation. These results reinforce the need to consider OPMDs in FA as distinct clinical conditions due to the unique characteristics of this population.

However, future studies should clearly distinguish OPMDs according to their specific subtypes, particularly in the context of chronic GvHD. This distinction requires histopathological confirmation, which was limited in the present study and represents an important limitation.

Longitudinal studies are essential to monitor the progression and possible malignant transformation of these lesions over time in individuals with FA. In this context, the present study should be considered a starting point for future subtype-specific and prospective research aimed at improving surveillance and clinical management strategies in this high-risk population.

## Declaration of generative AI and AI-assisted technologies in the manuscript preparation process

Generative AI and AI-assisted technologies were NOT used in the preparation of this work.

## Data availability

The data that support the findings of this study are available from the corresponding author upon reasonable request.

## Conflicts of interest

The authors of the article “Potentially Malignant Oral Disorders in Patients with Fanconi Anemia: A Cross-Sectional Study” declare that there is no conflict of interest.
